# Intra-pericardial Aneurism of Aorta

**Published:** 1871-12

**Authors:** 


INTRA-PERICARDIAL ANEURISM OF AORTA.—Dr. Stokes, Regius Professor of Physic in the University of Dublin, presented to the Pathological Society of Dublin (*Brit. Med. Jour.*, March 18, 1871,) a most interesting case illustrative of some obscure points in the diagnosis of cardiac disease. The patient was a man aged thirty-one, who six years ago was admitted to the Meath Hospital, suffering from "heart disease," most probably from pericarditis. At the time of his second admission, a short time ago, he was the subject of general anasarca. On physical examination, the liver was found to be much enlarged, its lower edge being felt just above the crest of the ilium. At its base a double murmur was audible, of which the first part was systolic, and the second corresponded with the diastole. This bruit became fainter when traced towards the apex, but at this point it was again distinctly heard. Besides the double basic murmur, a loud *frémissement* existed at the base. This sign disappeared at a subsequent period, but only to return. There was visible pulsation in the carotid alone, and the pulse partook to some extent of the characters of the collapsing form. Here, then, were all the usual signs of aortic patency, together with the basic *frémissement*, and a second double murmur at the apex. Dr. Hayden, who was asked by Dr. Stokes to see the case, suggested that the lesion was aneurism of the right ventricle. After death, the left ventricle proved to be much hypertrophied; the aortic valves were found perfectly competent, though somewhat thickened; and a true aneurism sprang just above the origin of the aorta. The tumor was intra-pericardial, and from the sac a fistulous passage led into the cavity of the right ventricle. There was, in fact, a varicose aneurism. The *frémissement* was now explained—its disappearance Dr. Stokes regarded as due to a temporary plugging of the fistulous openings. Cyanosis was never present, though before death the patient's aspect became unusually livid. Dr. Stokes mentioned that this was the second instance in his experience in which an aneurism springing in the neighborhood of the sinuses of Valsalva had perfectly simulated the comparatively common disease, permanent patency of the aortic valves. Dr. Hayden stated that his diagnosis was founded on the following considerations. First, the murmur of exit possessed a peculiar character, one never remarked in simple valvular disease. It resembled the sound caused by the entrance of fluid into a resounding cavity, and might best be described by the word "splashing." Secondly, this murmur was not transmitted into the carotid vessels.—*Am. Jour. Med. Sciences.*

